# Safety Evaluation and Imaging Properties of Gadolinium-Based Nanoparticles in nonhuman primates

**DOI:** 10.1038/srep35053

**Published:** 2016-10-11

**Authors:** Shady Kotb, Joao Piraquive, Franck Lamberton, François Lux, Michael Verset, Vanessa Di Cataldo, Hugues Contamin, Olivier Tillement, Emmanuelle Canet-Soulas, Lucie Sancey

**Affiliations:** 1 Univ Lyon, Institut Lumière Matière, UMR5306, Université Claude Bernard Lyon 1, CNRS, Institut Lumière Matière, F-69622, Villeurbanne, France; 2 Univ Lyon, CARMEN Laboratory, INSERM INSA, INRA, Université Claude Bernard Lyon 1, Lyon, France; 3 CERMEP, Imagerie du vivant, Bron 69677, France; 4 Cynbiose SAS, Marcy-L’Etoile, France

## Abstract

In this article, we report the safety evaluation of gadolinium-based nanoparticles in nonhuman primates (NHP) in the context of magnetic resonance imaging (MRI) studies in atherosclerosis bearing animals and healthy controls. In healthy NHP, the pharmacokinetics and toxicity profiles demonstrated the absence of dose, time, and sex-effects, as well as a suitable tolerance of intravenous administration of the nanoparticles. We investigated their imaging properties for arterial plaque imaging in a standard diet or a high cholesterol diet NHP, and compared their characteristics with clinically applied Gd-chelate. This preliminary investigation reports the efficient and safe imaging of atherosclerotic plaques.

Atherosclerosis is one of the main cardiovascular disorders resulting from an initial lipid accumulation in the artery wall with *in situ* lesion development as well as an unresolved chronic and complex inflammatory process^1^. This chronic and evolutive injury of the arterial wall may abruptly lead to the obstruction of the vessel itself by clot formation, or may lead to acute stroke following plaque rupture with cerebral emboli, which often leads to disastrous consequences^2^. Early imaging and monitoring of atherosclerosis and high-risk plaque is challenging as the lesion is non-obstructive and a precise non-invasive diagnosis might require the gathering of several parameters. As recent novel strategies are being developed for accurate detection, plaque burden can be measured using ultrasound exams or computed tomography (CT) for calcium scoring whereas macrophage infiltration and microcalcification can be monitored using a PET/CT or PET/MRI combination^3^,^4^. In parallel, high-resolution MRI allows for the depiction of angiogenesis, intraplaque hemorrhage, observation of necrotic core, or positive remodeling^5^,^6^,^7^,^8^. MRI can also be considered as the reference technique for vessel wall imaging and plaque characterization, especially for the carotid and peripheral arteries imaging^6^. A standard examination combines different high-resolution carotid T1-weighted and proton density-weighted, and a post-contrast agent T1-weighted acquisition^4^,^9^. For a better characterization of plaque microvasculature, dynamic contrast-enhanced (DCE) MRI is considered very helpful to identify leaky neovessels, a hallmark of plaque destabilization^10^.

Different contrast agents containing gadolinium in the chelated form are used for T1-weighted acquisitions. Commercially available gadolinium (Gd) chelates are molecular compounds containing one single Gd atom. Nevertheless, clustering several Gd chelates will enhance the relaxivity of the probe and thus, the related contrast imaging properties^11^,^12^. In this context, we used Gd-based nanoparticles for MRI purposes. Nanoparticles might allow the detection of atherosclerosis plaques^13^ or macrophages in inflammatory atherosclerosis^14^,^15^. We previously reported the efficient renal elimination (>50% of the injected dose at 74 min post injection)^16^ and the safety of Gd-based nanoparticles in rodents^17^,^18^, especially regarding the clearance mechanism^19^. Herein, we evaluated the safety and pharmacokinetics of Gd-based nanoparticles in healthy non-human primates (NHP). To further investigate the contrast potential, we reported their imaging properties in healthy and high cholesterol (HC) diet NHP and compared their characteristics to the vessel wall imaging with commercially available molecular Gd-chelate.

## Results

### Nanoparticles characteristics

Gd-NPs were produced using reported methods, in laboratory and GMP environment^16^,^20^. This nanoparticle regroups gadolinium (Gd)-chelating DOTAGA (1,4,7,10-tetra-azacyclododecane-1-glutaric anhydride-4,7,10-triacetic acid) coupled to a polysiloxane network. The hydrodynamic diameter of Gd-NPs is 3.5 ± 1 nm for a mass of ≈10 kDa. Due to the presence of Gd, the nanoparticle provides positive enhancement on T1-weighted MR images and radiosensitizing properties. Its imaging properties are investigated in addition to its safety profile in NHP under normal or high cholesterol (HC) diet.

### Toxicity and pharmacokinetic profile in nonhuman primates

Regulatory toxicity and pharmacokinetics were conducted in compliance with good laboratory practices (GLP), and were evaluated in nonhuman cynomolgus monkey primates at 3 different Gd-NP doses (low, 150 mg/kg body weight (b.w.); moderate, 300 mg/kg b.w.; and high, 450 mg/kg b.w.), during two-repeated injections protocol, *i.e*. once a week during two weeks. During this period, no cardiovascular or clinical signs were observed, neither in males nor females, at any dose (Table 1).

**Figure 1:**
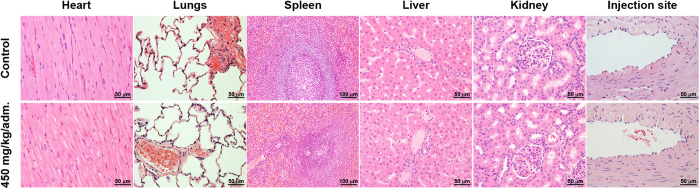
Examples of histological sections of vital organs and injection site of the control and the high-dose group. Hematoxylin and eosin staining revealed similar microscopic profiles when comparing control and high-dose group samples, in both males and females.

Two weeks after the last injection, all vital organs and injection sites were sampled for histological investigation. In all the tissues, no microscopic changes were evidenced after two administrations of Gd-NPs at a high dose of 450 mg/kg, compared to the control group (Fig. 1). In particular, the kidneys, which are the main organs of elimination, were similar to control kidneys, without any sign of vacuolation.

Plasma kinetics of Gd-NPs were evaluated for the treated groups after each administration, from 3 animals/sex/group, and are reported in Table 2. Blood samples were collected at 5 and 30 minutes, and 1, 2, 6, and 24 hours post-administration to determine the nanoparticles’ pharmacokinetics. Following the intravenous administration of Gd-NPs, the exposure in male and female cynomolgus monkeys increased in a dose-proportional manner for both sexes on both evaluation days. The exposure on day 7 was similar to that on day 0. The accumulation ratios ranged from 0.848 to 1.04 at all dose levels. On day 0, mean clearance as well as the distribution volume were low and ranged from 0.111 and 0.187 L/h/kg and 0.176 and 0.314 L/kg, respectively. The mean blood half-life (T_1/2_) ranged from 2.09 to 3.57 hours. In general, there were no trends observed related to dose, sex, or evaluation days for clearance, volume of distribution, or T_1/2_ values. Under these study conditions, two intravenous administrations at one-week interval of Gd-NPs at doses of 150, 300, and 450 mg/kg to the cynomolgus monkey were not associated with any overt evidence of intravenous toxicity. Consequently, the high dose (450 mg/kg/administration) could be considered to be the NOEL (non observed effect level). This dose corresponds to a mean area under the curve determined between 0 to 24 h (AUC_0–24h_) normalized to a unit dose (1 mg/kg b.w.) of 9.00/7.60 mg.h/mL (Day 0/Day 7) in males and of 6.42/6.32 mg.h/mL (Day 0/Day 7) in females.

### Imaging properties of the Gd-NPs in control (Cont) healthy monkeys

**Figure 2:**
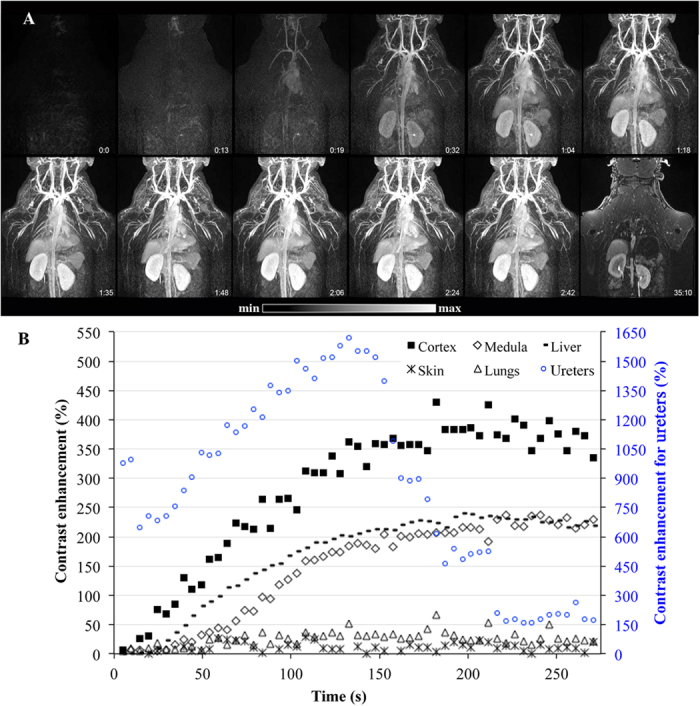
MRI first-pass kinetics of Gd-NPs in different tissues (liver, kidneys’ cortex and medulla, skin, and lungs) in a male control subject with a slow injection. (**A**) During the first minutes of the acquisition, Gd-NPs were administrated intravenously, allowing a clear observation of the blood network and main organs, such as the heart, liver, and kidneys, *i.e.* an excellent T1 enhancement for angiographic studies and fast renal excretion. Then, at 35 minutes, the kidneys and ureters were mainly observed, demonstrating the washout of the nanoparticles. (**B**) The contrast enhancement was determined on the main organs. The highest contrast enhancements were observed for the kidneys, liver, and ureters.

The T1-MRI properties of Gd-NPs were first studied in healthy monkeys to observe the general biodistribution of the particles. After the intravenous injection of Gd-NPs, the main vascular network was clearly identified, and the main organs, *i.e*. heart, liver, and kidneys. One should note that there was a marked enhancement of blood vessels at first-pass (Fig. 2, see also Figure S1) and the bolus injection was very well tolerated without any changes in hemodynamic, cardiac, or ventilation parameters. Within the first 30 minutes, most of the nanoparticles were eliminated by the kidney route, as observed in Fig. 2A (last panel). Low T1 contrast was persistent in the muscles, liver, and kidneys. The contrast enhancement indicated a rapid renal washout of the nanoparticles: the T1 contrast enhancement strongly increased in the ureters within the first 150 seconds, before it was drastically reduced during the next minute (Fig. 2B). Moreover, at 35 min post-administration, the T1 contrast of the ureters was once more very intense, indicating a continuous washout of Gd-NPs. The main MRI findings were in accordance with the pharmacokinetics’ profiles, which indicated a Gd-NPs blood half-life of ≈2 hours at the administrated dose (*i.e.* 200 mg/kg for MRI investigations).

### Imaging properties of Gd-NPs in old animals under a high cholesterol (HC) diet for 24 months

**Figure 3:**
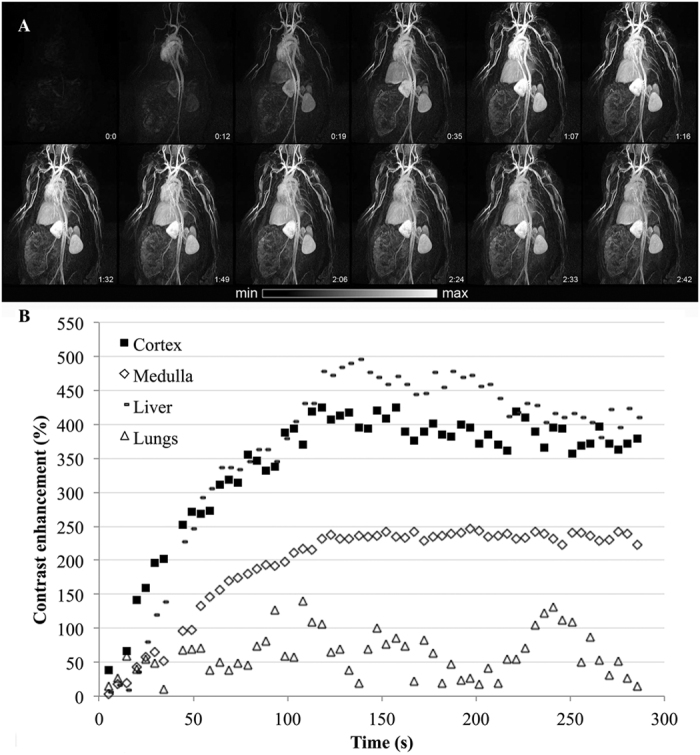
MRI first-pass kinetics of Gd-NPs in different tissues (liver, kidneys’ cortex and medulla, lungs) in a female HC subject with a slow injection. (**A**) Similar to healthy animals, Gd-NPs were distributed in the vascular network and the main organs, and rapidly reached the kidneys. (**B**) The contrast enhancements were determined for the kidneys, livers, and lungs.

Similarly to the previous investigation, MRI was performed on old monkeys under a 24-months HC diet (referred as HC^++^ animal, Fig. 3A). The contrast enhancements were similar to healthy animals except for the liver, which indicated a highest enhancement (Fig. 3B). Similar to healthy animals, the Gd-NPs were well tolerated without any changes in hemodynamic, cardiac or ventilation parameters.

### Contrast properties for vulnerable carotid plaque

**Figure 4:**
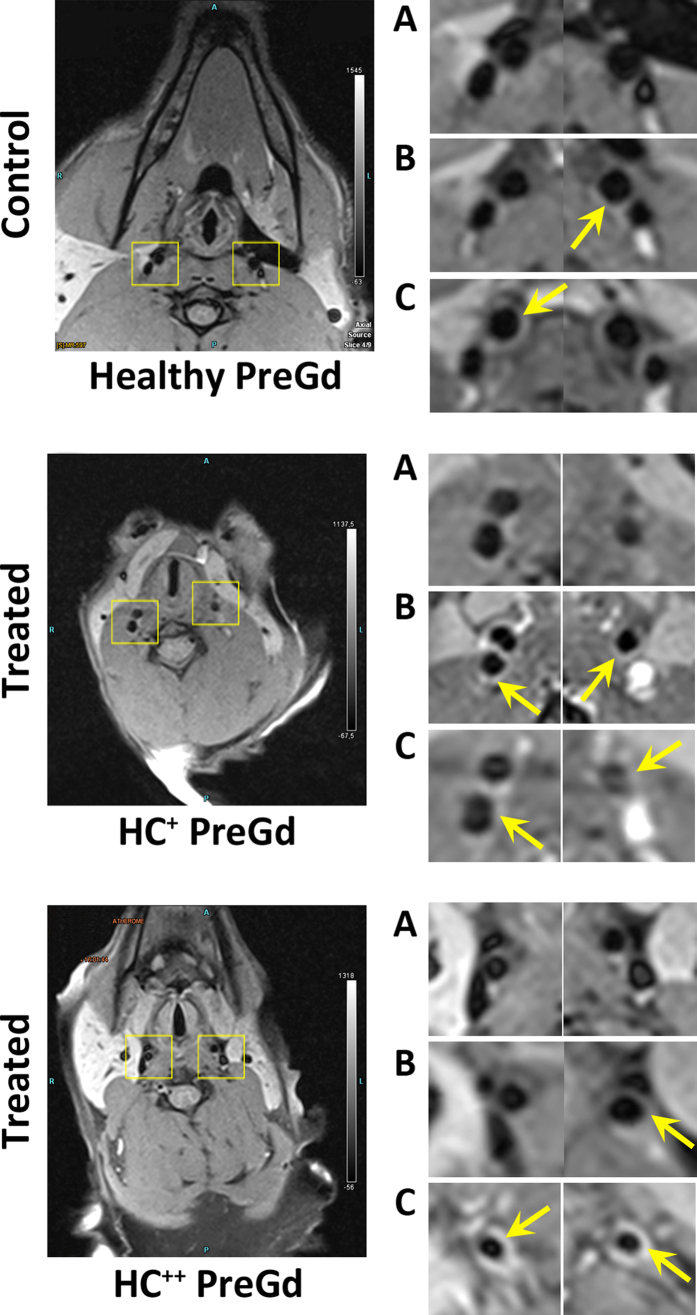
High-resolution vessel wall carotid MRI in control (upper panel) and HC animals (middle and lower panel). Enlarged views of the carotids (right panels) with pre-contrast T1 images (**A**), post-Gd-DOTA (**B**) and post-Gd-NPs (C) respectively. In HC animals, post-contrast enhancement of the vessel wall is characteristic of atherosclerotic lesions with inflammation and increased vessel wall permeability. Arrows: vessel wall.

**Figure 5:**
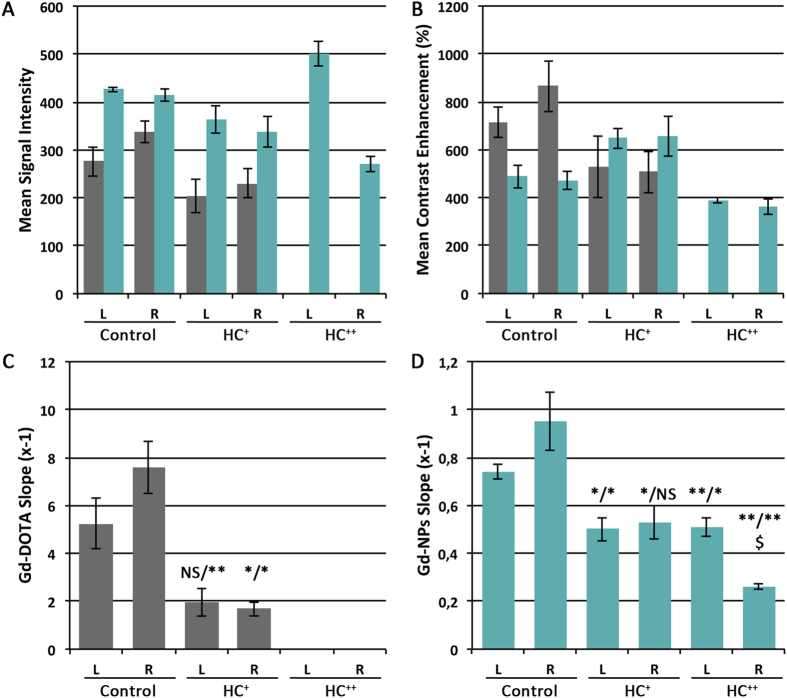
MRI characteristics of the left and right carotids after intravenous administrations of Gd-NPs. The uptake of Gd-NPs in the vulnerable carotid plaque was followed as function of time for signal intensity (**A**), and contrast enhancement (**B**). The calculated slopes of the washout were determined for Gd-DOTA (**C**, grey) and Gd-NPs (**D**, green). They were significantly different for vulnerable carotid plaques *versus* healthy carotids. L: left. R: right. HC: High cholesterol. For HC^++^ animal, Gd-DOTA values were not determined due to movements during the acquisition. *P < 0.05, **P < 0.01 HC *versus* healthy NHP. ^$^*p* < 0.05 for HC^++^ left *versus* right carotid.

The contrast properties of Gd-NPs were evaluated for vulnerable carotid plaques and compared to the ones of Gd-DOTA. In this pathology, unspecific accumulation of contrast agent may occur due to the leaky wall’s endothelial layer and the inflammation, which recruits highly active macrophages^21^. In our condition, the 24-months HC diet induced moderate and more advanced atherosclerosis, as indicated by the ultrasound and biochemical parameters recorded from the treated animals (Supplementary information Table S2). As indicated by pre-contrast T1 MRI (Fig. 4), the carotid walls were not observable before the administration of any contrast agent. In absence of vulnerable plaque, both Gd-DOTA and Gd-NPs allowed very minimal carotid wall delineation. In presence of vulnerable plaque, both Gd-DOTA and Gd-NPs delineated the carotid wall with similar contrast properties. Gd-NPs appeared to have similar imaging properties as compared to Gd-DOTA. In the case of a well-established pathology (Fig. 4, HC^++^), the vulnerable plaques were better identified by Gd-NPs, in comparison to Gd-DOTA. The T1-contrast obtained after Gd-NPs was measured with time and the elimination kinetics of Gd-NPs were determined (Fig. 5). In healthy animals, Gd-NPs were rapidly washed out, whereas a significant retention was observed in HC animals. Gd-NPs retention might be proportional with the stage of the pathology, as the highest retention was observed for the most developed pathology (right carotid of HC^++^ animal).

## Discussion – Conclusion

The use of nanoparticles as a contrast imaging agent requires their specific distribution in the body after intravenous injection, a rapid clearance from the body without undesired accumulation, a safe profile, and good contrast properties. Gd-NPs present the above mentioned properties with a fine distribution within the entire body starting at the first heartbeats following the intravenous administration, as well as a fast renal clearance as demonstrated with healthy NHP. After high-dose repeated IV administration, the particles were well tolerated, without modification of the antemortem and post-mortem parameters as compared to untreated animals. In particular, H&S staining indicated a safe renal elimination of Gd-NPs. Transient and minimal vacuolations of the proximal convoluted tubules was observed in rodents as previously reported^18^,^19^, but the NHP did not present such transient alteration for similar equivalent doses, indicating a strong tolerance and safe elimination in NHP. Mean blood half-life measured in NHP was very similar to the one measured in rats for equivalent doses, with 2.35 hours *versus* 2.31 hours, respectively^18^. Altogether, the safety profile indicated a NOEL of 450 mg/kg/administration, which corresponds to 145 mg/kg/administration for Humans^22^,^23^.

In contrast to intravascular ultrasound which accurately image the vessel wall at high resolution^24^, MRI is noninvasive. Combined to a T1-weighted contrast agent, MRI also allows to assess the morphological plaque characteristics. In particular, the local lesion and its evolution could be monitored using a carotid MRI protocol, considering the vessel wall permeability on gadolinium-enhanced MRI^5^. Under high cholesterol diet, old NHP had at-risk plasmatic profile (high LDL/HDL ratio, high triglycerides (see Table S3) and high hsCRP levels) and developped atherosclerosis lesions similar to human plaques at the same vascular sites^25^. As shown by MRI, our animals had carotid plaques with the same advanced and vulnerable characteristics as in patients.

For similar contrast properties^16^,^20^, Gd-DOTA is a cyclic ionic chelate, and Gd-NPs possess DOTA-derivatives. Both compounds possess very strong complexation for Gd (log*β*_110_ = 25.58 for Gd-DOTA, and log*β*_110_ = 25.58 for Gd-NPs), preventing the release of free Gd^20^. Safe administration was observed for old atherosclerosis NHP. Imaging of the vessel’s wall in this pathologic animal was demonstrated using both chelates and chelates bound to NPs. The signal measured in the vessel wall was correlated to the plaque development for Gd-NPs investigations; the former agent possesses a longer circulation time as compared to Gd-DOTA that is rapidly cleared from the body which may have favored its retention in the plaque (13.2 min *vs.* 6.8 min in mice, respectively)^19^. Therefore, this agent has more suitable properties to quantify neovessels leakiness using DCE-MRI, another important functional parameters to define vulnerable plaques^10^. Thanks to the high safety of Gd-NPs, this could be studied longitudinally in the near future using kinetic modeling in order to assess the vessel’s wall permeability over time and correlate it with the plaque’s evolution and downstream clinical events.

Altogether, this study demonstrates the safety of the Gd-NPs, in particular for old pathologic animals. The T1-contrast properties were previously reported for vessel wall and plaque imaging. Further studies will indicate the potential of Gd-NP for such investigations.

## Materiel and Methods

### Gadolinium-based nanoparticles

The gadolinium (Gd)-based nanoparticles (Gd-NP), AGuIX, were synthesized, purified, and characterized in compliance with GMP standards (Carbogen Amcis, Swiss). Gd-NPs are composed of an inorganic matrix of polysiloxane surrounded by covalently bound DOTAGA (Gd) ((1,4,7,10-tetra-azacyclododecane-1-glutaric anhydride - 4,7,10-triacetic acid) - Gd^3+^). The main chemical formula is Gd_10_Si_26_C_238_N_56_O_142_H_x_, for a 3.5 ± 1 nm hydrometric diameter and an overall charge of 9.5 ± 5.5 mV at pH 7.

### Nonhuman primate (NHP) studies

All animal studies and experiments were approved by the French Ministry of Agriculture and carried out in accordance with the official regulation of the French Ministry of Agriculture after approval by the local Ethical Committee (No 1367 & 1239).

### Pharmacokinetics and toxicity study

A total of 24 healthy cynomolgus monkeys (*macaca fascicularis*) (n = 3/sex/group) were assigned to 4 groups: control, low-, moderate-, and high-dose of AGuIX series, *i.e.* 0, 150, 300, and 450 mg/kg/administration, respectively (Wil Research, France). The doses were administered intravenously, once per week for 2 weeks (day 0 and day 7). Blood samples were collected following each administration at 5 and 30 min, 1, 2, 6 and 24 h. The blood plasma distribution kinetics was analyzed based on a non-compartment model (Kinetica 4.4.1, Thermo Fisher). All animals were observed for mortality, clinical signs, ophthalmology, body weight, food consumption, hematology, biochemistry, pathology, toxicokinetics, and urinary parameters. Animals from the control group received a sterile solution of pure water containing calcium chloride (1.5 mM) and NaOH to mimic the adjuvant at pH 7.4. Hematoxylin and eosin (HES) staining were performed on tissue sections excised from the heart, lung, kidneys and liver to visualize the toxicity induced by AGuIX, 2 weeks after the second administration.

### Atherosclerosis NHP model

Atherosclerosis animals were evaluated after being 24 months under a high cholesterol diet. The model is developed by Cynbiose (Marcy L’Etoile, France), and the animals of the present study are part of a larger study to evaluate plaque vulnerability using imaging biomarkers in this translational NHP model. Briefly, after ovariectomy, old females were fed a high cholesterol (HC) diet (Energy 19.3 MJ/kg, with 45 kJ% from fat, containing 0.5% cholesterol, 23% lipids, 11.3% from saturated fatty acids and 38% sugars, mainly sucrose) (V3944-000, Ssniff, Germany). The food ration was adapted according to the animal’s body weight (100 g for animals under 5 kg and 200 g for animals over 5 kg). The study duration was 24 months. One fruit was provided daily to each animal. Delicacies were also occasionally given to the animals at the end of the day as part of the Testing Facility environmental enrichment program. The control animals were maintained under the same housing conditions, but with the regular NHP diet (Energy 13.7 MJ/kg, with 51 kJ% from carbohydrate, and 11 kJ% from fat containing 4% fat, and 10.2% sugars) (Ssniff, Germany). Total cholesterol and lipidic profiles were evaluated in plasma and high sensitive C reactive protein (hsCRP) was measured in serum. Expert cardiologist and veterinarian under general anesthesia performed ultrasonography, at 12 and 18 months to evaluate plaque development in different vascular beds: the carotids, aorta, and iliac arteries. Gene expression and cytokines were measured in the heart, carotids, and aorta at the end of the atherosclerosis inflammatory profile study.

### Magnetic Resonance Imaging

The biodistribution study was carried out in cynomolgus monkeys under a normal diet (*macaca fascicularis,* control animals 5 and 8 y.o., n = 2) and in high cholesterol fed NHPs (HC^+/++^ animals 16 and 17 y.o., n = 2) at 200 mg/mL of AGuIX *i.e*. 0.1 mmol of Gd/kg equiv. Magnetic resonance imaging (MRI) was performed with a 3T scanner (Magnetom Prisma, Siemens, Erlangen, Germany) equipped with high performance gradients (80 mT/m, slew rate 200 T/m/s). All images were acquired with a set of standard multi-channels received MRI coils delivered by the manufacturer. These coils were installed once during the positioning of the animal in the scanner and activated according to the field of view. First, the monkeys were installed in supine position on the lower part of the 64-channel Head/Neck coil as well as the 32-channel Spine coil. Then two additional 4-channel flexible surface coils were placed on top on the neck (SC coil, size of 14 cm by 9 cm) and the thorax (FS coil, size of 37 cm by 7 cm) respectively. Whole body and high resolution imaging of the carotids were performed under general anesthesia (xylazine/ketamine, 100–200 mg/kg) with continuous monitoring of temperature, O_2_ saturation and cardiac/respiration rate. The induction was performed by intramuscular injections of ketamine at 10 mg/kg (Ketamine 1000, Virbac, Carros, France) and midazolam at 0.2 mg/kg (Midazolam Aguettant, Lyon, France) followed by anesthetic maintenance by intravenous infusion of ketamine at 12 mg/kg/h. Dynamic images were acquired with the TWIST (Time resolved angiography With Interleaved Stochastic Trajectories) pulse sequences. TWIST sequence was applied in the coronal orientation and acquired with a separation of 5 seconds between frames. The imaging parameters were: TR/TE/Flip angle = 2.95 msec/1.10 msec/25° and voxel size 0.8 × 0.8 × 1.2 mm^3^. For comparison, a bolus of gadolinium chelate (0.1 mmol Gd/kg, Gd-DOTA, DOTAREM® Guerbet, Aulnay-sous-Bois, France) was administered intravenously at 0.1 mL/sec followed by a saline flush. After renal excretion of standard gadolinium chelate, the sequence was repeated with the Gd-NPs at an adjusted dose for a similar angiographic T1 effect (0.1 mmol Gd/kg Gd-NPs). High-resolution images of the carotids were acquired with an ECG-gated Turbo Spin Echo sequence with black-blood module. The parameters were TR/TE = 466 ms/5.8 ms, TSE factor = 9, reconstructed voxel size of 0.3 × 0.3 × 2 mm and 9 slices planed perpendicular to the common carotid using a TOF acquisition.

### MRI data treatment

MRI data analysis was performed using the Inveon® Research Workplace 4.1 software (Siemens, Erlangen, Germany). The regions of interest were drawn in the carotid vessel wall and in the artery’s lumen to obtain the dynamic contrast information during the contrast agent first-pass, and in the different organs of interest for the contrast distribution at a steady state.

### Statistical analysis

Statistical analysis was performed using unpaired T-Test (Excel software) for the vulnerable plaque contrast.

## Additional Information

**How to cite this article**: Kotb, S. *et al*. Safety Evaluation and Imaging Properties of Gadolinium-Based Nanoparticles in nonhuman primates. *Sci. Rep.* **6**, 35053; doi: 10.1038/srep35053 (2016).

## Supplementary Material

Supplementary Information

## Figures and Tables

**Table 1:** List of the investigations performed after 2-repeated IV injection of Gd-NPs in cynomolgus monkey.

In life parameters	Safety Pharmacology	Clinical Pathology	Terminal parameters
Mortality/Morbidity	Electrocardiogram^a^	Haematology	Necropsy
Clinical signs	Blood pressure^b^	Coagulation	HistoPathology
Local tolerance	Respiratory rate	Serum clinical chemistry	
Body weight	Neurobehavioral assessment	Urinary analysis	
Food consumption			
Ophtalmology			

^a^Includes heart rate, QRS complex duration, PR intervals, QT intervals.

^b^Includes diastole and systole. No differences were reported at any dose. Details can be found in the Supplementary information Table S1.

**Table 2:** Mean pharmacokinetic parameters in male and female cynomolgus monkeys following two intravenous administrations of Gd-NPs.

		Dose (mg/kg/day)	AUC_0–24h_	DN	C_max_	DN	T_1/2_	Cl	V_ss_	Acc. Ratio
Day, Gender			(h*mg/mL)	AUC_0-24h_	(mg/mL)	C_max_	(h)	(L/h/kg)	(L/kg)	
Day 0	M	150	812	5.41	1,429	9.53	2.13	0.187	0.195	
	M	300	1,866	6.22	3,305	11.02	2.17	0.162	0.176	
	M	450	4,051	9.00	4,792	10.65	3.57	0.111	0.314	
	F	150	884	5.89	1,422	9.48	2.21	0.171	0.225	
	F	300	1,695	5.65	3,216	10.72	2.09	0.178	0.184	
	F	450	2,890	6.42	4,541	10.09	2.34	0.150	0.185	
Day 7	M	150	828	5.52	1,437	9.58	2.29	NA	NA	1.02
	M	300	1,934	6.45	3,198	10.66	2.22	NA	NA	1.04
	M	450	3,419	7.60	4,779	10.62	NA	NA	NA	0.848
	F	150	831	5.54	1,528	10.19	2.30	NA	NA	0.948
	F	300	1,665	5.55	2,984	9.95	2.19	NA	NA	0.983
	F	450	2,842	6.32	4,979	11.06	NA	NA	NA	0.991

M: Male; F: Female; AUC: Area under the curve; DN: Dose-Normalized; C_max_: Maximum plasma concentration; T½: blood half-life; Cl: Clearance; V_ss_: Volume of distribution at the steady state.Units for DN AUC_0–24h_ is (mg*h/mL)/(mg/kg) and units for DN C_max_ is (mg/mL)/(mg/kg). Acc. Ratio = Accumulation Ratio. The values were determined at 5 and 30 minutes, 1, 2, 6, and 24 hours post-administration.

## References

[b1] Yahagi K. . Pathophysiology of native coronary, vein graft, and in-stent atherosclerosis. Nat Rev Cardiol 13, 79–98, doi: 10.1038/nrcardio.2015.164 (2016). 26503410

[b2] Libby P. Mechanisms of acute coronary syndromes and their implications for therapy. The New England journal of medicine 368, 2004–2013, doi: 10.1056/NEJMra1216063 (2013). 23697515

[b3] Adamson P. D., Vesey A. T., Joshi N. V., Newby D. E. & Dweck M. R. Salt in the wound: (18)F-fluoride positron emission tomography for identification of vulnerable coronary plaques. Cardiovasc Diagn Ther 5, 150–155, doi: 10.3978/j.issn.2223-3652.2015.03.01 (2015). 25984456 PMC4420673

[b4] Dweck M. R., Puntman V., Vesey A. T., Fayad Z. A. & Nagel E. M. R. Imaging of Coronary Arteries and Plaques. JACC Cardiovasc Imaging 9, 306–316, doi: 10.1016/j.jcmg.2015.12.003 (2016). 26965732

[b5] Canet-Soulas E. & Letourneur D. Biomarkers of atherosclerosis and the potential of MRI for the diagnosis of vulnerable plaque. Magma 20, 129–142, doi: 10.1007/s10334-007-0078-y (2007). 17605060

[b6] Kramer C. M. & Anderson J. D. MRI of atherosclerosis: diagnosis and monitoring therapy. Expert Rev Cardiovasc Ther 5, 69–80, doi: 10.1586/14779072.5.1.69 (2007). 17187458 PMC3938864

[b7] Moody A. R., Allder S., Lennox G., Gladman J. & Fentem P. Direct magnetic resonance imaging of carotid artery thrombus in acute stroke. Lancet 353, 122–123 (1999). 10023906 10.1016/s0140-6736(05)76159-6

[b8] Murphy R. E. . Prevalence of complicated carotid atheroma as detected by magnetic resonance direct thrombus imaging in patients with suspected carotid artery stenosis and previous acute cerebral ischemia. Circulation 107, 3053–3058, doi: 10.1161/01.CIR.0000074204.92443.37 (2003). 12796136

[b9] Dweck M. R. . Imaging of coronary atherosclerosis - evolution towards new treatment strategies. Nat Rev Cardiol, doi: 10.1038/nrcardio.2016.79 (2016). 27226154

[b10] van Hoof R. H., Heeneman S., Wildberger J. E. & Kooi M. E. Dynamic Contrast-Enhanced MRI to Study Atherosclerotic Plaque Microvasculature. Curr Atheroscler Rep 18, 33, doi: 10.1007/s11883-016-0583-4 (2016). 27115144 PMC4846686

[b11] Fries P. . Evaluation of a Gadolinium-Based Nanoparticle (AGuIX) for Contrast-Enhanced MRI of the Liver in a Rat Model of Hepatic Colorectal Cancer Metastases at 9.4 Tesla. RoFo: Fortschritte auf dem Gebiete der Rontgenstrahlen und der Nuklearmedizin 187, 1108–1115, doi: 10.1055/s-0035-1553500 (2015). 26361379

[b12] Lux F. . Gadolinium-based nanoparticles for theranostic MRI-radiosensitization. Nanomedicine 10, 1801–1815, doi: 10.2217/nnm.15.30 (2015). 25715316

[b13] Lobatto M. E. . Atherosclerotic plaque targeting mechanism of long-circulating nanoparticles established by multimodal imaging. ACS nano 9, 1837–1847, doi: 10.1021/nn506750r (2015). 25619964 PMC4492477

[b14] Majmudar M. D. . Polymeric nanoparticle PET/MR imaging allows macrophage detection in atherosclerotic plaques. Circ Res 112, 755–761, doi: 10.1161/CIRCRESAHA.111.300576 (2013). 23300273 PMC3586287

[b15] Bagalkot V. . Hybrid nanoparticles improve targeting to inflammatory macrophages through phagocytic signals. J Control Release 217, 243–255, doi: 10.1016/j.jconrel.2015.09.027 (2015). 26386437 PMC4874242

[b16] Lux F. . Ultrasmall rigid particles as multimodal probes for medical applications. Angew Chem Int Ed Engl 50, 12299–12303, doi: 10.1002/anie.201104104 (2011). 22057640

[b17] Detappe A. . Advanced multimodal nanoparticles delay tumor progression with clinical radiation therapy. J Control Release 238, 103–113, doi: 10.1016/j.jconrel.2016.07.021 (2016). 27423325

[b18] Verry C. . MRI-guided clinical 6-MV radiosensitization of glioma using a unique gadolinium-based nanoparticles injection. Nanomedicine, doi: 10.2217/nnm-2016-0203 (2016). 27529506

[b19] Sancey L. . Long-term *in vivo* clearance of gadolinium-based AGuIX nanoparticles and their biocompatibility after systemic injection. ACS nano 9, 2477–2488, doi: 10.1021/acsnano.5b00552 (2015). 25703068

[b20] Mignot A. . A top-down synthesis route to ultrasmall multifunctional Gd-based silica nanoparticles for theranostic applications. Chemistry 19, 6122–6136, doi: 10.1002/chem.201203003 (2013). 23512788

[b21] Ronald J. A. . Comparison of gadofluorine-M and Gd-DTPA for noninvasive staging of atherosclerotic plaque stability using MRI. Circ Cardiovasc Imaging 2, 226–234, doi: 10.1161/CIRCIMAGING.108.826826 (2009). 19808597 PMC2759097

[b22] Blanchard O. L. & Smoliga J. M. Translating dosages from animal models to human clinical trials--revisiting body surface area scaling. FASEB J 29, 1629–1634, doi: 10.1096/fj.14-269043 (2015). 25657112

[b23] Reagan-Shaw S., Nihal M. & Ahmad N. Dose translation from animal to human studies revisited. FASEB J 22, 659–661, doi: 10.1096/fj.07-9574LSF (2008). 17942826

[b24] Celeng C., Takx R. A., Ferencik M. & Maurovich-Horvat P. Non-invasive and invasive imaging of vulnerable coronary plaque. Trends Cardiovasc Med, doi: 10.1016/j.tcm.2016.03.005 (2016). 27079893

[b25] Di Cataldo V. . Mr and pet/ct imaging use to stratify cardiovascular risks in non-human primates under atherogenic diet. Atherosclerosis 241, E161–E161 (2015).

