# Supplementary material for: Safety Evaluation and Imaging Properties of Gadolinium-Based Nanoparticles in nonhuman primates
Source: Sci Rep. 2016 Oct 11;6:35053. doi: 10.1038/srep35053 (PMC5057154; doi:10.1038/srep35053)
Supplement: Supplementary Information [file srep35053-s1.doc]

Safety Evaluation and Imaging Properties of Gadolinium-Based Nanoparticles in nonhuman primates

Authors:

Kotb Shady^1$^, Piraquive Joao^2$^, Lamberton Franck^3^, Lux François^1^, Verset Michael^4^, Di Cataldo Vanessa^2^, Contamin Hugues^4^, Tillement Olivier^1^, Canet-Soulas Emmanuelle^2^, Sancey Lucie^1*^

Supplementary information:

***Table S1 In vivo*** safety pharmacology studies in Cynomolgus Monkeys: detailed data on Blood Pressure, Heart Rate, and ECG before and after treatment of Gd-NPs

| Before treatment (Day -9) | | | | | | | |
| --- | --- | --- | --- | --- | --- | --- | --- |
| Group | Electrocardiogram | | | | Blood pressure | | Respiratory rate (breaths  /min) |
|  | Heart Rate (beats/min) | QRS complex duration (ms) | PR interval (ms) | QT intervals (ms) | Diastolic (mm Hg) | Systolic (mm Hg) |  |
| M-0 | 246.7 ± 15.3 | 76.7 ± 3.5 | 60.3 ± 5.8 | 172.3 ± 4.0 | 89 ± 12.2 | 163.7 ± 20.4 | 49.0 ± 3.6 |
| M-150 | 256.7 ± 15.3 | 78.7 ± 5.1 | 54.3 ± 2.3 | 174.3 ± 19.6 | 102 ± 8.5 | 179.3 ± 9 | 48.0 ± 2.6 |
| M-300 | 253.3 ± 20.8 | 77.7 ± 4.0 | 58.0 ± 1.7 | 172.0 ± 1.7 | 93.3 ± 6.1 | 156.3 ± 13.3 | 41.3 ±2.9 |
| M-450 | 266.7 ± 15.3 | 76.6 ± 6.5 | 51.3 ± 5.1 | 165.3 ± 6.8 | 88.7 ± 5.1 | 147 ± 22.6 | 48.3 ± 2.9 |
| F-0 | 243.3 ± 20.8 | 78.0 ± 1.7 | 56.7 ± 3.5 | 175.7 ± 5.1 | 94.7 ± 10.2 | 159.3 ± 13.6 | 56.0 ± 5.2 |
| F-150 | 223.3 ± 32.1 | 75.7 ± 5.1 | 61.0 ± 1.7 | 177.7 ± 13.6 | 99.7 ± 14 | 168.3 ± 27 | 52.0 ± 3.5 |
| F-300 | 250 ± 10 | 74.3 ± 5.1 | 59.0 ±3.5 | 177.7 ± 6.8 | 100.3 ± 2.5 | 170.7 ± 15.6 | 55.3 ± 6.7 |
| F-450 | 240 ± 10 | 81.0 ± 3.5 | 55.7 ± 7.5 | 192.3 ± 6.8 | 97 ± 10.1 | 171 ± 14.9 | 49.7 ± 4.7 |
|  |  |  |  |  |  |  |  |
| Before treatment (Day -9) | | | | | | | |
| Group | Electrocardiogram | | | | Blood pressure | | Respiratory rate (breaths  /min) |
|  | Heart Rate (beats/min) | QRS complex duration (ms) | PR interval (ms) | QT intervals (ms) | Diastolic (mm Hg) | Systolic (mm Hg) |  |
| M-0 | 226.7 ± 51.3 | 84.3 ± 7.5 | 62.0 ± 11.5 | 190.0 ± 28.8 | 100 ± 5.3 | 177.3 ± 6.4 | 46.7 ± 11.6 |
| M-150 | 250 ± 10 | 77.7 ± 5.0 | 57.0 ± 0.0 | 175.7 ± 18.0 | 102.7 ± 23.4 | 185.7 ± 36.1 | 53.0 ± 7.8 |
| M-300 | 230 ± 17.3 | 79.0 ± 1.7 | 64.3 ± 7.5 | 185.7 ± 5.1 | 90.3 ± 12.1 | 158 ± 25.6 | 47.0 ± 5.2 |
| M-450 | 260 ± 17.3 | 71.3 ± 5.1 | 55.3 ± 4.0 | 180.0 ± 13.0 | 97 ± 15.5 | 180.3 ± 11 | 44.0 ± 1.0 |
| F-0 | 263.3 ± 5.8 | 73.0 ± 0.0 | 51.0 ± 8.5 | 167.7 ± 10.8 | 96 ± 10.8 | 163.3 ± 20.4 | 47.7 ± 5.5 |
| F-150 | 223.3 ± 25.2 | 79.0 ± 1.7 | 63.3 ± 5.8 | 181.3 ± 5.1 | 101 ± 12.1 | 172 ± 13 | 48.7 ± 12.5 |
| F-300 | 263.3 ± 15.3 | 75.7 ± 2.3 | 54.7 ± 4.0 | 174.7 ± 13.7 | 97.7 ± 8.1 | 174.7 ± 13.3 | 46.3 ± 4.2 |
| F-450 | 246.7 ± 25.2 | 79.0 ± 1.7 | 58.7 ± 9.8 | 189.0 ± 18.5 | 102.7 ± 5.7 | 182.7 ± 7 | 40.7 ± 6.4 |

**Example of MR imaging in NHP:**


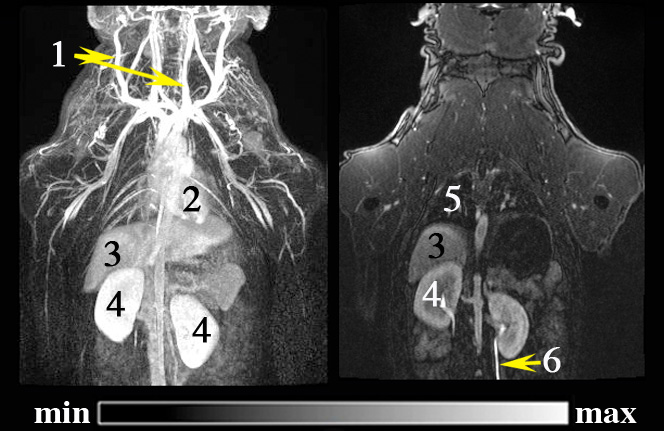


**Figure S1**: Example of MR Imaging of Gd-NPs in healthy NHP. 1: Main vascular network; 2: Heart; 3: Liver; 4: Kidneys; 5: Lungs; 6: Ureters.

**Atherosclerosis characteristics:**

The two old HC NHP included in this study (respectively 16 and 17 years old) had increased total cholesterol (respectively, 402 and 386 mg/dL compared to 100 mg/dL in normal NHP), and high LDL (220 and 258 mg/dL compared to 50 mg/dL in normal NHP). The two ultrasonography exams showed evolving atherosclerosis as at least two vascular beds (iliac arteries and carotids) showed lesion progression between 12 and 18 months. At the last time point, the inflammatory plasma profiles (high TNFalpha, respectively 125 and 157 pg/mL *versus* 38 pg/mL in normal NHP) were in agreement with increased cytokines in the heart, aortic arch, and carotids (increased IL1-beta and IL6 production, >0.2 pg/mg and >1 pg/mg of proteins respectively), further assessed by increased inflammatory macrophage gene expressions in the same tissues.

***Table S2 Main atherosclerosis characteristics of the NHP (HC+ with moderate carotid plaque evolution between the two ultrasound exams, HC^++^ more advanced lesions)***

| NHP | Age (y.o.) | Total cholesterol (mg/dL) | High LDL (mg/dL) | hsCRP  (µg/mL of serum) | TNFalpha (pg/mL) | IL production (pg/mg of proteins) |
| --- | --- | --- | --- | --- | --- | --- |
| Control | 8 | 100 | 50 | 18 | 38 | Low |
| HC^+^ | 16 | 402 | 220 | 38 | 125 | >0.2 |
| HC^++^ | 17 | 386 | 258 | 55 | 157 | >1 |

***Table S3 Serum clinical chemistry parameters. Liver and Kidney enzymes were measured at 12-months of diet.*** ALT: Alanine Amino-Transferase, AST: Aspartate Amino-Transferase, CREAT: creatinine, GGT: Gamma Glutamyl-Transferase, ALP: alkaline phosphatase, Trigs: Triglycerides, *elevated values.

| NHP | ALT  (U/L) | AST  (U/L) | CREAT  (µmol/L) | GGT  (U/L) | Glucose  (mmol/L) | ALP  (U/L) | Trigs  mg/dL |
| --- | --- | --- | --- | --- | --- | --- | --- |
| Control | 68 | 50 | 71 | 150 | 2.2 | 460 | 54 |
| HC^+^ | 48 | 93* | 105* | 115 | 3.4 | 204 | 229* |
| HC^++^ | 56 | 61* | 93* | 142 | 3.2 | 248 | 262* |
